# Clinical and genetic characteristics of patients with early-onset or familial parkinsonism: A retrospective single-center cohort

**DOI:** 10.1371/journal.pone.0361198

**Published:** 2026-10-27

**Authors:** Jiannan Chen, Qi Hu, Shanshan Wei, Dongxuan Zhang, Zhe Zhao

**Affiliations:** 1 Department of Neurology, Hebei Medical University Third Hospital, Shijiazhuang, Hebei, People’s Republic of China; 2 Department of Gastroenterology, Beijing Changping Hospital of Traditional Chinese Medicine, The Affiliated Changping Hospital of Beijing University of Chinese Medicine, Beijing, People’s Republic of China; MGI Tech Co., Ltd., CHINA

## Abstract

**Background:**

Parkinson’s disease (PD) is the second most prevalent neurodegenerative disorder after Alzheimer’s disease. Hereditary causes account for 10–15% of cases but present considerably more complex clinical manifestations than idiopathic cases. It is therefore imperative to enhance clinicians’ recognition of hereditary forms.

**Methods:**

This study enrolled 29 patients with early‑onset or familial parkinsonism. 17 cases were identified with reportable genetic findings using PD-targeted or movement disorders gene panels. Clinical data and genetic data were collected.

**Results:**

Among 17 patients, PARK2/*PRKN* (n = 9) and PARK8/*LRRK2* (n = 5) were predominant subtypes, with two cases of PARK14/*PLA2G6*, and one case of PARK18/*EIF4G1*. Clinically, bradykinesia affected 17/17 patients, rigidity 15/17, tremor 13/17, and dystonia 7/17 (including one oculogyric crisis). Hyperreflexia occurred in 6/17, cognitive impairment in 2/17, pyramidal signs in 2/17, ataxic dysarthria 1/17, and pes cavus in 1/17. Diurnal fluctuation and sleep benefit occurred exclusively in PARK2/*PRKN* and PARK14/*PLA2G6* patients. Among patients with *PRKN*, 7/9 exhibited diurnal fluctuations: five had exon rearrangements, one had a splicing mutation, and one had a homozygous variant. Conversely, the two PARK2/*PRKN* patients without diurnal fluctuations showed no exon deletions/duplications. PARK18/*EIF4G1* mutations may manifest incomplete penetrance.

**Conclusion:**

Among PARK2/*PRKN* patients in our cohort, a potential association between diurnal fluctuation and exon rearrangements may exist, although this observation requires cautious interpretation. Furthermore, these variants appear to cluster within the N-terminal region of the protein (the start of the RING1 domain and preceding regions). Beyond the common PARK2/*PRKN* subtype, PARK14/*PLA2G6* may also be linked to diurnal fluctuation and sleep benefit.

## Introduction

Parkinson’s disease (PD) is the second most prevalent neurodegenerative disorder after Alzheimer’s disease (AD) [1], affecting approximately 1% of individuals aged over 60 years and 4% of those over 85 years worldwide [2,3]. The clinical diagnosis of PD is primarily based on a combination of typical motor and nonmotor symptoms, including bradykinesia, tremor and rigidity, anosmia, sleep disorders, and autonomic abnormalities [4,5]. However, the low initial diagnostic accuracy of 58% severely hinders early intervention and disease management [6]. While most PD cases are sporadic, approximately 10%–15% of all PD patients and 25% of early-onset PD (EOPD) cases have a clear familial genetic background, indicating that genetic variation is a core pathogenic factor for PD, especially for early-onset and atypical PD subtypes [7,8].

Genetic studies have confirmed that multiple pathogenic genes with different inheritance patterns are closely associated with PD occurrence, and significant genotype-phenotype heterogeneity widely exists in genetic PD [9]. Existing studies have demonstrated that different PD gene mutations correspond to distinct clinical manifestations, disease progression patterns, and neuropathological features. Over 90% of genetically-related phenotypes are complex or atypical, with dystonia, abnormal cognition, pyramidal signs, neuropsychiatric disorders, abnormal imaging, and abnormal eye movements [10]. For instance, *LRRK2* mutations usually induce typical idiopathic PD-like phenotypes [11], while *SNCA* mutations cause highly variable clinical spectrums ranging from typical EOPD to dementia with Lewy bodies [9]. Mutations in *PRKN* and *PINK1* mostly lead to slowly progressive EOPD, whereas other recessive gene variants tend to cause severe atypical parkinsonism with complex clinical phenotypes [9]. In the Chinese population, the mutational spectrum of EOPD is dominated by *PRKN*, *GBA*, and *LRRK2* mutations [12–14].

Marked diurnal fluctuation and sleep benefit, alongside a marked and sustained response to low-dose levodopa, are considered hallmark features of dopa-responsive dystonia (DRD) [15]. However, Yamamura et al. first described autosomal recessive familial cases presenting with early-onset parkinsonism accompanied by prominent diurnal fluctuation and sleep benefit as early as 1973 [16]. Symptoms are relatively mild in the morning and deteriorate in the late afternoon or evening, a pattern that differs from classic PD, in which symptoms worsen in the morning. Sleep benefit is a relatively common phenomenon: previous studies have reported that approximately 45% of patients exhibit sleep benefit after waking [17]. Although this phenomenon has been studied for decades, and multiple factors have been proposed to correlate with sleep benefit in earlier investigations, these associations remain controversial [18].

Against this background, the present study enrolled a cohort of 17 Chinese patients with early-onset or familial parkinsonism and conducted a retrospective observational study. The core research objectives were as follows: (1) to clarify the genetic subtype distribution and summarize the phenotypic characteristics of the enrolled patients; (2) to screen and identify previously unreported PD pathogenic variants in the Chinese population; (3) to further explore the genotype–phenotype correlations of hereditary parkinsonism in this local cohort. This study aims to supplement the clinical genetic data of PD in the Chinese population and provide additional observational evidence for genotype–phenotype correlations, especially for the phenotypes of diurnal fluctuation and sleep benefit.

## Methods

### Patients

Patients with clinically diagnosed early‑onset or familial parkinsonism who underwent genetic testing at the Department of Neurology, the Hebei Medical University Third Hospital between January 1, 2016 and June 30, 2025, and who had not received anti‑parkinsonian treatment were retrospectively enrolled. Demographic and clinical data, including sex, age, family history, past medical history (medication use and exposure to heavy metals or other toxic substances), current illness, neurological examination (performed by at least two specialist neurologists), routine laboratory tests and cranial MRI, were extracted from the electronic medical record system. The study protocol was approved by the Ethics Committee of Hebei Medical University Third Hospital (ethics approval number: W2025-058-1). The research was conducted in accordance with the Declaration of Helsinki. Informed consent was obtained from all subjects and/or their legal guardians.

Inclusion criteria: Patients presenting with parkinsonism should fulfill one of the following conditions: age at onset ≤50 years, or age at onset ≥50 years with a positive family history (i.e., at least one first or second degree relative diagnosed with Parkinson’s disease or manifesting parkinsonism), together with a favorable/partial response to levodopa benserazide (≥15% improvement in MDS UPDRS III motor score) [19].

Based on the 2015 MDS‑PD diagnostic criteria [20], parkinsonism is defined as bradykinesia, in combination with either rest tremor, rigidity, or both. Bradykinesia is defined as slowness of movement and decrement in amplitude or speed (or progressive hesitations/halts) as movements are continued. Bradykinesia can be evaluated by using finger tapping (MDS-UPDRS 3.4), hand movements (3.5), pronation-supination movements (3.6), toe tapping (3.7), and foot tapping (3.8). Rigidity refers to “leadpipe” resistance. Rest tremor refers to a 4- to 6-Hz tremor in the fully resting limb, which is suppressed during movement initiation.

Exclusion criteria: Patients with identifiable etiologies underlying secondary parkinsonism were excluded, including those with exposure to specific medications (e.g., chlorpromazine, haloperidol, risperidone, olanzapine, metoclopramide, flunarizine, cinnarizine); toxin exposure (e.g., carbon monoxide, manganese, pesticides, organic solvents); recurrent stroke; infections (e.g., encephalitis, syphilis, HIV); inflammatory demyelinating disorders; and metabolic disorders (e.g., thyroid or parathyroid dysfunction, Wilson’s disease).

### Gene testing

Two distinct sequencing panels were adopted in this study: a comprehensive PD-targeted panel (covering next-generation sequencing (NGS) for point mutations, MLPA, and repeat expansion screening) and a movement disorder (MD) panel (integrating NGS, capture-based CNV (Cap-CNV) analysis, and repeat expansion screening). The MD panel provides broader gene coverage. Based on the PD-targeted sequencing framework, it additionally includes genes responsible for hereditary spastic paraplegia, ataxia, peripheral neuropathy, leukoencephalopathy, and other related neurological disorders. The gene lists for the PD-targeted panel and the MD panel are provided in S1 Table and S2 Table, respectively.

**DNA Library Preparation**: Genomic DNA was extracted from peripheral blood samples obtained from patients and their parents using the QIAamp DNA Mini Kit (Qiagen, Shanghai, China), following the manufacturer’s instructions. Standard libraries were constructed with the DNA Sample Prep Reagent Set (MyGenostics, Beijing, China) and included end repair, adapter ligation, and PCR amplification. The constructed libraries were sequenced on an Illumina NovaSeq 6000 sequencer (Illumina, San Diego, CA, USA). **Enrichment and Sequencing of Targeted Genes**: Amplified DNA libraries were captured using the targeted gene capture panel (MyGenostics Inc., Beijing, China). Biotinylated 100-bp capture probes were designed to tile across all coding exons plus 50 bp flanking regions of target genes. Capture was performed per the manufacturer’s protocol. Raw sequencing was performed using paired-end reads of 2 × 150 bp. The mean sequencing depth for target regions was at least 300×, and the proportions of target bases covered at ≥10× and ≥20 × were at least 98% and 96%, respectively. **Bioinformatics Analysis**: Raw sequencing data were stored in FASTQ format. MGI sequencing adapters and low-quality reads (<80 bp) were trimmed using Cutadapt (http://code.google.com/p/cutadapt/). Clean reads were mapped to the UCSC hg19 human reference genome using the BWA module within Sentieon software (https://www.sentieon.com/). Duplicate reads were removed, and base quality recalibration was performed with Sentieon. Base recalibration adjusts base quality scores to better reflect the true probability of read-to-reference mismatches. Mapped reads were used for variant calling. SNPs and InDels were detected using Sentieon; variant data were converted to VCF format. Variants were annotated with ANNOVAR (http://annovar.openbioinformatics.org/en/latest/) against multiple databases, including the 1000 Genomes Project, ESP6500, dbSNP, ExAC, an in-house MyGenostics database, and HGMD. Functional predictions were generated using SIFT, PolyPhen-2, MutationTaster, and GERP++. **Variant Selection**: Benign and likely benign variants were excluded per American College of Medical Genetics and Genomics (ACMG) guidelines [21]. For quality control, retained variants were required to meet MutCount ≥10 and MutRatio ≥0.3; variants with insufficient sequencing support were discarded. Variants annotated as pathogenic in HGMD, ClinVar, and the MyGenostics internal case database were retained. Variants present in public population databases (1000G, ESP6500) and our in-house healthy control database were excluded. Synonymous variants lacking pathogenic evidence in HGMD were excluded, as were noncoding variants without HGMD-documented pathogenicity. Priority was assigned to exonic and splice-site variants. Population allele frequency thresholds were applied according to inheritance patterns: MAF ≤ 0.02 for recessive disorders and MAF ≤ 0.001 for dominant disorders. Remaining variants were further curated by integrating disease inheritance patterns and patients’ clinical phenotypes to identify candidate pathogenic variants. Sanger sequencing was performed to validate all candidate pathogenic variants.

## Results

A total of 29 cases meeting enrollment criteria were collected, with 17 demonstrating positive genetic findings and 12 negative. The negative group had a mean age at onset of 36.6 years; two patients were under 20 years, while seven were over 40 years old. Among 17 patients, PARK2/*PRKN* (n = 9) and PARK8/*LRRK2* (n = 5) were predominant, with two cases of PARK14/*PLA2G6*, and one case of PARK18/*EIF4G1* (Clinical and genetic data of the 17 patients are summarized in Table 1 and Table 2). No *GBA* mutations were detected despite their reported high frequency in Asian populations [12]. Clinically, bradykinesia affected 17/17 patients, rigidity 15/17, tremor 13/17, and dystonia 7/17 (including one oculogyric crisis). Hyperreflexia occurred in 6/17, cognitive impairment in 2/17, pyramidal signs in 2/17, ataxic dysarthria 1/17, and pes cavus in 1/17. Three patients with combined tremor had both resting and postural tremor (Patient 5, 6, and 17).

**Table 1:** Clinical data of the 17 patients.

Case	Sex	AAO/Age	Clinical manifestations				Diurnal fluctuation	Gene/Subtype
			Tremor	Bradykinesia	Rigidity	Others		
1	F	18/25	**+**	**+**	**+**	Dystonia, Hyperreflexia	**+**	PARK2
2	M	33/33	**+**	**+**	**+**	-	**+**	PARK2
3	M	19/23	**+**	**+**	**+**	Dystonia, Pyramidal signs	**+**	PARK2
4	F	27/33	**+**	**+**	**+**	Pes cavus, Hyperreflexia	**+**	PARK2
5	F	13/23	**+**	**+**	**+**	Dystonia, Hyperreflexia	**+**	PARK2
6	M	13/33	**+**	**+**	**-**	Dystonia, ataxic dysarthria	**+**	PARK2
7	M	52/53	**-**	**+**	**+**	-	**+**	PARK2
8	M	29/31	**-**	**+**	**+**	-	**-**	PARK2
9	M	23/24	**-**	**+**	**+**	Dystonia, Cognitive impairment	**-**	PARK2
10	M	36/37	**+**	**+**	**+**	-	**-**	PARK8
11	M	49/49	**+**	**+**	**-**	-	**-**	PARK8
12	F	48/53	**+**	**+**	**+**	Hyperreflexia	**-**	PARK8
13	F	41/43	**-**	**+**	**+**	Hyperreflexia, Cognitive impairment	**-**	PARK8
14	F	59/60	**-**	**+**	**+**	-	**-**	PARK8
15	F	27/27	**+**	**+**	**+**	Oculogyric crisis	**+**	PARK14
16	F	40/40	**+**	**+**	**+**	Dystonia, Hyperreflexia, Pyramidal signs	**-**	PARK14
17	M	47/48	**+**	**+**	**+**	-	**-**	PARK18

F: female; M: male. AAO: age at onset; Age: current age. **+** : positive; **-**: negative.

**Table 2:** Genetic data of the 17 patients.

Case	Sex	AAO/Age	Gene/Subtype	Variation 1	Variation 2	Inheritance
1	F	18/25	*PRKN*	c.850G > C, p.G284R (P)	c.850G > C, p.G284R(P)	AR
2	M	33/33	*PRKN*	exon3 del (P)	exon2-4 del (P)	AR
3	M	19/23	*PRKN*	exon1 del (P)	exon 5-6 dup (P)	AR
4	F	27/33	*PRKN*	c.2T > C, p.M1T (LP)	c.619-3C > G, splicing (LP)	AR
5	F	13/23	*PRKN*	exon6 del (P)	exon6 del (P)	AR
6	M	13/33	*PRKN*	exon4 del (P)	c.2T > C, p.M1T (LP)	AR
7	M	52/53	*PRKN*	exon3 del (P)	c.2T > C, p.M1T (LP)	AR
8	M	29/31	*PRKN*	c.1063_1064insACCA,p.G355Dfs*16 (P)	c.850G > C, p.G284R (P)	AR
9	M	23/24	*PRKN*	c.1372A > C, p.M458L (VUS)^a^	-	AD
10	M	36/37	*LRRK2*	c.3200G > A, p.R1067Q (LP)	-	AD
11	M	49/49	*LRRK2*	c.1256C > T, p.A419V(VUS) ^a^	-	AD
12	F	48/53	*LRRK2*	c.3200G > A, p.R1067Q (LP)	-	AD
13	F	41/43	*LRRK2*	c.7153G > A, p.G2385R(VUS) ^a, b^	-	AD
14	F	59/60	*LRRK2*	**c.4303C > T, p.L1435F** (VUS)^b^	-	AD
15	F	27/27	*PLA2G6*	c.1894C > T, p.R632W (P)	**c.967G > A, p.V323M** (VUS)	AR
16	F	40/40	*PLA2G6*	**c.1060C > G, p.L354V** (VUS)	**c.1219A > G, p.A407G** (VUS)	AR
17	M	47/48	*EIF4G1*	c.4319C > T, p.S1440F (VUS) ^a^	-	AD

F: female; M: male. AAO: age at onset; P: pathogenic; LP: likely pathogenic; VUS: variants of uncertain significance; AD: autosomal dominant; AR: autosomal recessive. a: Despite being a VUS, this variant has been reported to be associated with PD. b: Despite being a VUS, this variant is carried by the relatives with concordant clinical manifestations. Note: The variants highlighted in bold are novel.

Among patients in this cohort, onset age in PARK2/*PRKN* group (mean: 25.2 years; range: 13–52 years) was earlier than in PARK8/*LRRK2* group (mean: 46.6 years; range: 36–59 years). Dystonia occurred in 5/9 PARK2/*PRKN* patients, cognitive decline in 1/9, and pyramidal signs in 1/9. In PARK8/*LRRK2*, all patients except one with cognitive decline exhibited a phenotype consistent with idiopathic PD. Diurnal fluctuation (non-medication-related) and sleep benefit were observed in PARK2/*PRKN* and PARK14/*PLA2G6*. Among PARK2/*PRKN* patients, 7/9 exhibited these features: five had exon rearrangements, one had a splicing mutation, and one had a homozygous variant. Conversely, the two without diurnal fluctuation showed no exon deletions or duplications (Schematic diagram of the Parkin protein and the mutations identified in this study are shown in Fig 1). The male-to-female ratio was 4:3. Although Patient 9 met the inclusion criteria, this patient exhibited a relatively complex phenotype, including cognitive decline and dystonia. Of note, the improvement rate was between 15% and 30% in Patient 9 during the levodopa challenge test, whereas all other patients in this cohort achieved an improvement rate >30%. The reported variant in Patient 18/PARK18 was maternally inherited, yet his mother exhibited normal phenotypes, suggesting possible incomplete penetrance. Incomplete penetrance of PD caused by *EIF4G1* variants has been documented in familial cases, indicating involvement of other genetic or environmental factors [22]. The Patient 15 with *PLA2G6* presented with oculogyric crisis, a form of dystonia. Thus, the patient was diagnosed with dystonia-parkinsonism. However, no iron deposition was observed on cranial MRI. Although the clinical phenotype of PARK14/*PLA2G6* is complex [23], diurnal fluctuation and sleep benefit remain unreported.

**Fig 1:**
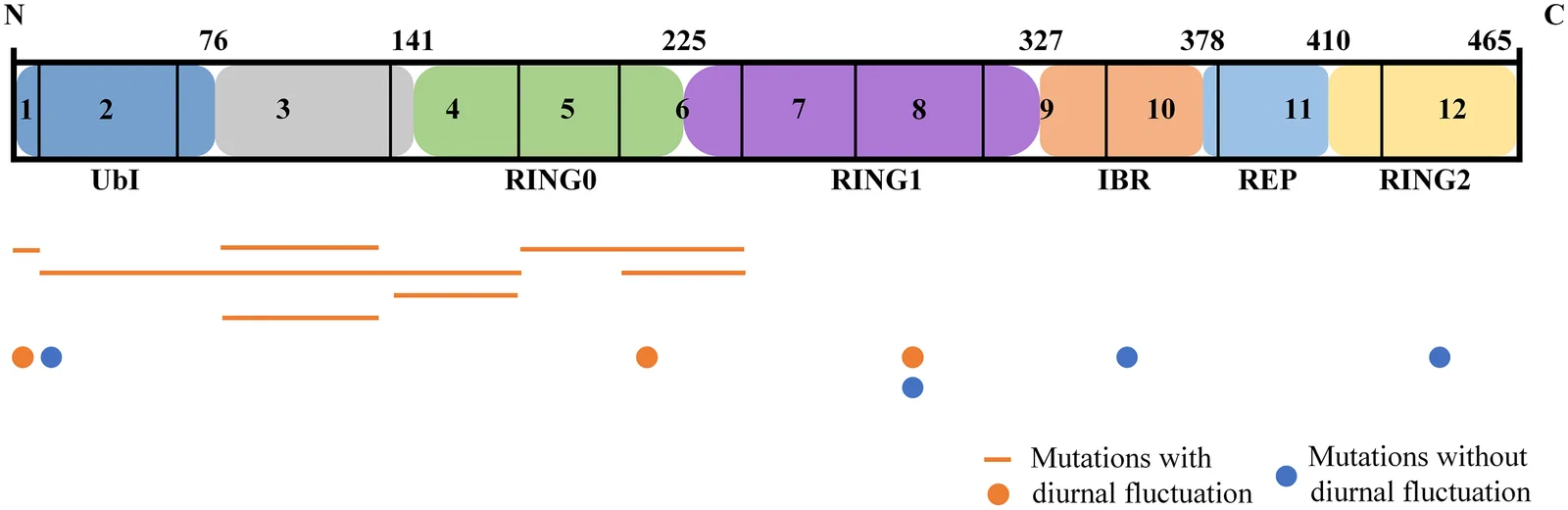
Schematic diagram of the Parkin protein and the mutations identified (Horizontal lines indicate exon rearrangements, and dots indicate missense mutations, splicing mutations, and frameshift mutations).

## Discussion

### Diurnal fluctuation and sleep benefit in hereditary parkinsonism

Sleep benefit in PD denotes improved motor symptoms upon awakening after a medication-free night. Yamamura et al. (1973) [24] first described ‘paralysis agitans of early onset with marked diurnal fluctuation’. Subsequent reports identified similar features predominantly in juvenile PD (JPD, onset ≤21 years), frequently associated with *PRKN*, *PINK1*, and *DJ-1* mutations [12]. Sleep benefit occurs in up to 55% of such patients, lasting several hours and sometimes following daytime naps [25,26]. Although proposed several decades ago, this phenomenon remains difficult to investigate, with its true prevalence and mechanisms still unclear. Physiological explanations have been hypothesized, favoring replenishment of dopaminergic stores in nigral terminals during sleep [25–28]. Traditionally, diurnal fluctuation, sleep benefit, and a dramatic, sustained levodopa response are hallmark features of DRD [15,29]. Adult-onset DRD may present with focal dystonia and parkinsonism [29]. Thus, distinguishing JPD from DRD based on atypical symptoms can be challenging. In DRD, diurnal fluctuation is prominent in early childhood but diminishes during the second decade, attributed to tetrahydrobiopterin (BH4) short half-life and circadian oscillation of tyrosine hydroxylase activity at dopaminergic terminals in younger patients (activity decreases during wakefulness and increases during rest) [30,31]. However, patients with *PRKN* in the study who exhibited significant diurnal fluctuation persisted into their third or fourth decades. In a study of 43 autosomal recessive-EOPD patients, marked diurnal fluctuation occurred in 41 cases (95.3%), absent only in the two with onset after age 40, while most patients reported even 30–40 years post-onset [32]. Additionally, parkinsonism with diurnal fluctuation is also documented in other hereditary disorders, including ataxia telangiectasia, spinocerebellar ataxia type (SCA) 3, hereditary spastic paraplegia type 8 and type 11 [33–36].

Interestingly, in the study, 5 of 7 PARK2/*PRKN* patients exhibiting diurnal fluctuation carried exon rearrangement mutations. In contrast, no exon rearrangements were detected in patients without such fluctuations. Furthermore, these variants were predominantly clustered in the N-terminal region of the protein (the start of the RING1 domain and preceding regions). The core functions of the N-terminal region of the Parkin protein up to the RING1 domain-including the Ubl domain, linker region, and RING0-are to maintain an autoinhibited resting state, mediate PINK1-dependent activation, and act as a molecular switch regulating E3 ubiquitin ligase activity. The RING1 domain and the subsequent C-terminal domains, including IBR, REP, and RING2, constitute the catalytic core of the Parkin protein. It is possible that such phenotypic heterogeneity is attributable to differential functional impairment. Accordingly, large-scale studies are still needed to perform comprehensive statistical analyses.

In a separate study reported by Yamamura et al. [32], they investigated 95.3% of their cohort exhibited diurnal fluctuations. Among 37 patients from 16 families who underwent genetic testing, no pathogenic variants were identified in 16 patients from 5 families, while exon rearrangements were detected in the remaining patients. These findings may suggest a potential association between diurnal fluctuation and *PRKN* exon rearrangements, although this observation requires cautious interpretation.

DRD exhibits distinct gender predilection compared with other disorders, with an incidence two to three times higher in females than males. Among cases of hereditary parkinsonism with diurnal fluctuation, reported sex differences are inconsistent: one study identified sleep benefit to be more prevalent in males (65%) than females (42%) [28]. Both Zhong Rui et al. [18] and Yamamura et al. [32] detected no significant sex differences in PD patients with diurnal fluctuation. Nevertheless, the meta-analysis by Zhong Rui et al. demonstrated lower sleep efficiency in patients presenting sleep benefit, a finding that challenges the classical hypothesis of dopaminergic store replenishment within nigral terminals during sleep. However, most participants included in that meta-analysis had sporadic PD, and several received relevant pharmacotherapy. Thus, the influencing factors and mechanisms underlying sleep benefit in untreated hereditary parkinsonism warrant further investigation.

### PARK2/*PRKN*

*PRKN* is the most common cause of autosomal recessive early-onset parkinsonism [37,38]. Bradykinesia is the most common sign, followed by tremor (especially in the legs), rigidity, dystonia, and postural instability [39]. Atypical features such as anterocollis, spasticity, and upper motor neuron signs, as well as cognitive decline, each occur in only about 3% of cases [39]. Although bradykinesia is the most common initial sign of *PRKN*- associated parkinsonism, one report found that 42% of patients reported dystonia as an initial sign [40]. In the PARK2/*PRKN* patients of this study, pyramidal signs, cognitive decline, pes cavus and ataxic dysarthria were observed. *PRKN* mutations have been reported in some cases of progressive supranuclear palsy, restless legs syndrome, and essential tremor [41]. Notably, one patient with a heterozygous *PRKN* variant (likely pathogenic), who was excluded from the final cohort of this study, exhibited isolated postural tremor responsive to alcohol, a feature consistent with essential tremor. His mother had similar symptoms and harbored the identical pathogenic variant. Nevertheless, a causal link between *PRKN* variants and essential tremor cannot be established based on this single case. Furthermore, it remains unclear whether this phenotype represents a preclinical or early stage of PD, which warrants long-term clinical follow-up.

Notably, although *PRKN* mutations were traditionally presumed to follow autosomal recessive inheritance, increasing evidence suggested that heterozygous mutations might also be pathogenic or increase susceptibility to typical late-onset PD [42–44]. Patient 9, with a heterozygous variant that has previously been reported to be associated with PD [45], exhibited a complex parkinsonism phenotype with a partial response to levodopa. Additionally, whole-exome sequencing performed previously at an external hospital revealed no additional pathogenic variants in this patient. Although autosomal dominant inheritance has been considered, this case warrants further exploration and validation.

### PARK8/*LRRK2*

While the motor symptoms of *LRRK2*-related parkinsonism are largely indistinguishable from sporadic PD, significant differences exist in age at onset (AAO), disease penetrance, and neuropathology [8]. Neuropathology ranges from nigrostriatal degeneration with variable Lewy body and Alzheimer’s pathology to absence of these features or atypical findings [8]. *LRRK2* carriers also typically exhibit better olfactory function, less frequent rapid eye movement sleep behavior disorder (RBD), and diverse atypical features including orthostatic hypotension, dementia, and hallucinations [3]. *LRRK2* parkinsonism generally progresses slower. In this study, all PARK8 patients presented with IPD except one case accompanied by cognitive decline. One patient had an onset age of 59 years; genetic testing was prompted by a family history of PD. Otherwise, patients at this age would usually not be considered for genetic screening.

In summary, our findings may advance the understanding of this disorder. Among PARK2/*PRKN* patients in our cohort, a potential association between diurnal fluctuation and exon rearrangements may exist, although this observation requires cautious interpretation. Furthermore, these variants appear to cluster within the N-terminal region of the protein (the start of the RING1 domain and preceding regions). Beyond the common PARK2/*PRKN* subtype, PARK14/*PLA2G6* may also be linked to diurnal fluctuation and sleep benefit. Nevertheless, several limitations of this study should be noted. As a single-center retrospective investigation with a small sample size, selection bias may be present, especially concerning missed *GBA* variant detection. Moreover, a subset of patients was analyzed using a movement disorder gene panel. While the panel contained cap-CNV analysis, it is less sensitive than MLPA for large *GBA* rearrangements, potentially contributing to undetected *GBA* variants. In addition, diurnal fluctuations and sleep benefit were assessed via patient self-report without quantitative measurements.

## Supporting information

S1 Table Gene list of the PD-targeted panel. (DOCX)

S2 Table Gene list of the Movement disorder (MD) panel. (DOCX)

## References

[1] Erkkinen MG, Kim M-O, Geschwind MD. Clinical neurology and epidemiology of the major neurodegenerative diseases. Cold Spring Harb Perspect Biol. 2018;10(4):a033118. doi: 10.1101/cshperspect.a033118 28716886 PMC5880171

[2] Deng H, Gao K, Jankovic J. The VPS35 gene and Parkinson’s disease. Mov Disord. 2013;28(5):569–75. doi: 10.1002/mds.25430 23536430

[3] Deng H, Wang P, Jankovic J. The genetics of Parkinson disease. Ageing Res Rev. 2018;42:72–85. doi: 10.1016/j.arr.2017.12.007 29288112

[4] Michael TH. Parkinson’s disease and parkinsonism. Am J Med. 2019;132:802–7. doi: 10.1016/j.amjmed.2019.03.001 30890425

[5] Postuma RB, Poewe W, Litvan I, Lewis S, Lang AE, Halliday G, et al. Validation of the MDS clinical diagnostic criteria for Parkinson’s disease. Mov Disord. 2018;33(10):1601–8. doi: 10.1002/mds.27362 30145797

[6] Beach TG, Adler CH. Importance of low diagnostic accuracy for early Parkinson’s disease. Mov Disord. 2018;33(10):1551–4. doi: 10.1002/mds.27485 30288780 PMC6544441

[7] Sellbach AN, Boyle RS, Silburn PA, Mellick GD. Parkinson’s disease and family history. Parkinsonism Relat Disord. 2006;12(7):399–409. doi: 10.1016/j.parkreldis.2006.03.002 16797215

[8] Chittoor-Vinod VG, Nichols RJ, Schüle B. Genetic and environmental factors influence the pleomorphy of LRRK2 parkinsonism. Int J Mol Sci. 2021;22(3):1045. doi: 10.3390/ijms22031045 33494262 PMC7864502

[9] Lunati A, Lesage S, Brice A. The genetic landscape of Parkinson’s disease. Rev Neurol (Paris). 2018;174(9):628–43. doi: 10.1016/j.neurol.2018.08.004 30245141

[10] Morales-Briceño H, Mohammad SS, Post B, Fois AF, Dale RC, Tchan M, et al. Clinical and neuroimaging phenotypes of genetic parkinsonism from infancy to adolescence. Brain. 2020;143(3):751–70. doi: 10.1093/brain/awz345 31800013

[11] Thaler A, Ash E, Gan-Or Z, Orr-Urtreger A, Giladi N. The LRRK2 G2019S mutation as the cause of Parkinson’s disease in Ashkenazi Jews. J Neural Transm (Vienna). 2009;116(11):1473–82. doi: 10.1007/s00702-009-0303-0 19756366

[12] Zhao Y, Qin L, Pan H, Liu Z, Jiang L, He Y, et al. The role of genetics in Parkinson’s disease: A large cohort study in Chinese mainland population. Brain. 2020;143(7):2220–34. doi: 10.1093/brain/awaa167 32613234

[13] Chen Y, Gu X, Ou R, Zhang L, Hou Y, Liu K, et al. Evaluating the Role of SNCA, LRRK2, and GBA in Chinese patients with early-onset Parkinson’s disease. Mov Disord. 2020;35(11):2046–55. doi: 10.1002/mds.28191 32677286

[14] Lin C-H, Chen P-L, Tai C-H, Lin H-I, Chen C-S, Chen M-L, et al. A clinical and genetic study of early-onset and familial parkinsonism in taiwan: An integrated approach combining gene dosage analysis and next-generation sequencing. Mov Disord. 2019;34(4):506–15. doi: 10.1002/mds.27633 30788857 PMC6594087

[15] Chen Y, Bao X, Wen Y, Wang J, Zhang Q, Yan J. Clinical and genetic heterogeneity in a cohort of Chinese children with dopa-responsive dystonia. Front Pediatr. 2020;8:83. doi: 10.3389/fped.2020.00083 32185155 PMC7058807

[16] Matsumine H, Yamamura Y, Kobayashi T, Nakamura S, Kuzuhara S, Mizuno Y. Early onset parkinsonism with diurnal fluctuation maps to a locus for juvenile parkinsonism. Neurology. 1998;50(5):1340–5. doi: 10.1212/wnl.50.5.1340 9595984

[17] van Gilst MM, Louter M, Baumann CR, Bloem BR, Overeem S. Sleep benefit in Parkinson’s disease: time to revive an enigma? J Parkinsons Dis. 2012;2:167–70. doi: 10.3233/JPD-2012-12087 23939441

[18] Rui Z, Qingling C, Xinyue Z, Xin Z, Weihong L. The related factors of sleep benefit in Parkinson’s disease: A systematic review and meta-analysis. PLoS One. 2019;14(3):e0212951. doi: 10.1371/journal.pone.0212951 30856190 PMC6411108

[19] Goetz CG, Tilley BC, Shaftman SR, Stebbins GT, Fahn S, Martinez-Martin P, et al. Movement disorder society-sponsored revision of the unified Parkinson’s Disease Rating Scale (MDS-UPDRS): Scale presentation and clinimetric testing results. Mov Disord. 2008;23(15):2129–70. doi: 10.1002/mds.22340 19025984

[20] Postuma RB, Berg D, Stern M, Poewe W, Olanow CW, Oertel W, et al. MDS clinical diagnostic criteria for Parkinson’s disease. Mov Disord. 2015;30(12):1591–601. doi: 10.1002/mds.26424 26474316

[21] Richards S, Aziz N, Bale S, Bick D, Das S, Gastier-Foster J, et al. Standards and guidelines for the interpretation of sequence variants: A joint consensus recommendation of the American College of Medical Genetics and Genomics and the Association for Molecular Pathology. Genet Med. 2015;17(5):405–24. doi: 10.1038/gim.2015.30 25741868 PMC4544753

[22] Nuytemans K, Bademci G, Inchausti V, Dressen A, Kinnamon DD, Mehta A, et al. Whole exome sequencing of rare variants in EIF4G1 and VPS35 in Parkinson disease. Neurology. 2013;80(11):982–9. doi: 10.1212/WNL.0b013e31828727d4 23408866 PMC3653206

[23] Karkheiran S, Shahidi GA, Walker RH, Paisán-Ruiz C. PLA2G6-associated dystonia-Parkinsonism: Case report and literature review. Tremor Other Hyperkinet Mov (N Y). 2015;5:317. doi: 10.7916/D84Q7T4W 26196026 PMC4503963

[24] Yamamura Y, Sobue I, Ando K, Iida M, Yanagi T. Paralysis agitans of early onset with marked diurnal fluctuation of symptoms. Neurology. 1973;23(3):239–44. doi: 10.1212/wnl.23.3.239 4735177

[25] van Gilst MM, Bloem BR, Overeem S. “Sleep benefit” in Parkinson’s disease: A systematic review. Parkinsonism Relat Disord. 2013;19(7):654–9. doi: 10.1016/j.parkreldis.2013.03.014 23615667

[26] Lee W, Evans AH, Williams DR. Objective measurement and characterization of sleep benefit in Parkinson’s disease. Mov Disord Clin Pract. 2017;4(4):590–6. doi: 10.1002/mdc3.12489 30363502 PMC6174513

[27] Tandberg E, Larsen JP, Karlsen K. Excessive daytime sleepiness and sleep benefit in Parkinson’s disease: A community-based study. Mov Disord. 1999;14(6):922–7. doi: 10.1002/1531-8257(199911)14:6<922::aid-mds1003>3.0.co;2-7 10584665

[28] Merello M, Hughes A, Colosimo C, Hoffman M, Starkstein S, Leiguarda R. Sleep benefit in Parkinson’s disease. Mov Disord. 1997;12(4):506–8. doi: 10.1002/mds.870120405 9251067

[29] Lee W-W, Jeon BS. Clinical spectrum of dopa-responsive dystonia and related disorders. Curr Neurol Neurosci Rep. 2014;14(7):461. doi: 10.1007/s11910-014-0461-9 24844652 PMC4061475

[30] Segawa M. Hereditary progressive dystonia with marked diurnal fluctuation. Brain Dev. 2011;33(3):195–201. doi: 10.1016/j.braindev.2010.10.015 21094587

[31] Nomura Y, Uetake K, Yukishita S, Hagiwara H, Tanaka T, Tanaka R, et al. Dystonias responding to levodopa and failure in biopterin metabolism. Adv Neurol. 1998;78:253–66. 9750922

[32] Yamamura Y, Hattori N, Matsumine H, Kuzuhara S, Mizuno Y. Autosomal recessive early-onset parkinsonism with diurnal fluctuation: clinicopathologic characteristics and molecular genetic identification. Brain Dev. 2000;22 Suppl 1:S87–91. doi: 10.1016/s0387-7604(00)00130-3 10984666

[33] Charlesworth G, Mohire MD, Schneider SA, Stamelou M, Wood NW, Bhatia KP. Ataxia telangiectasia presenting as dopa-responsive cervical dystonia. Neurology. 2013;81(13):1148–51. doi: 10.1212/WNL.0b013e3182a55fa2 23946315 PMC3795596

[34] Wilder-Smith E, Tan EK, Law HY, Zhao Y, Ng I, Wong MC. Spinocerebellar ataxia type 3 presenting as an L-DOPA responsive dystonia phenotype in a Chinese family. J Neurol Sci. 2003;213(1–2):25–8. doi: 10.1016/s0022-510x(03)00129-1 12873751

[35] Wijemanne S, Shulman JM, Jimenez-Shahed J, Curry D, Jankovic J. SPG11 mutations associated with a complex phenotype resembling dopa-responsive dystonia. Mov Disord Clin Pract. 2015;2(2):149–54. doi: 10.1002/mdc3.12144 30363882 PMC6183009

[36] Mulroy E, Magrinelli F, Mohd Fauzi NA, Kutty SK, Latorre A, Bhatia KP. Paroxysmal, exercise-induced, diurnally fluctuating dystonia: Expanding the phenotype of SPG8. Parkinsonism Relat Disord. 2021;85:26–8. doi: 10.1016/j.parkreldis.2021.02.011 33662919

[37] Ferreira M, Massano J. An updated review of Parkinson’s disease genetics and clinicopathological correlations. Acta Neurol Scand. 2017;135(3):273–84. doi: 10.1111/ane.12616 27273099

[38] Deng H, Le W-D, Hunter CB, Ondo WG, Guo Y, Xie W-J, et al. Heterogeneous phenotype in a family with compound heterozygous parkin gene mutations. Arch Neurol. 2006;63(2):273–7. doi: 10.1001/archneur.63.2.273 16476817

[39] Schneider SA, Alcalay RN. Neuropathology of genetic synucleinopathies with parkinsonism: Review of the literature. Mov Disord. 2017;32(11):1504–23. doi: 10.1002/mds.27193 29124790 PMC5726430

[40] Lücking CB, Dürr A, Bonifati V, Vaughan J, De Michele G, Gasser T, et al. Association between early-onset Parkinson’s disease and mutations in the parkin gene. N Engl J Med. 2000;342(21):1560–7. doi: 10.1056/NEJM200005253422103 10824074

[41] Niemann N, Jankovic J. Juvenile parkinsonism: Differential diagnosis, genetics, and treatment. Parkinsonism Relat Disord. 2019;67:74–89. doi: 10.1016/j.parkreldis.2019.06.025 31272925

[42] Kobayashi T, Matsumine H, Zhang J, Imamichi Y, Mizuno Y, Hattori N. Pseudo-autosomal dominant inheritance of PARK2: Two families with parkin gene mutations. J Neurol Sci. 2003;207(1–2):11–7. doi: 10.1016/s0022-510x(02)00358-1 12614925

[43] Poorkaj P, Nutt JG, James D, Gancher S, Bird TD, Steinbart E, et al. parkin mutation analysis in clinic patients with early-onset Parkinson [corrected] disease. Am J Med Genet A. 2004;129A(1):44–50. doi: 10.1002/ajmg.a.30157 15266615

[44] Foroud T, Uniacke SK, Liu L, Pankratz N, Rudolph A, Halter C, et al. Heterozygosity for a mutation in the parkin gene leads to later onset Parkinson disease. Neurology. 2003;60(5):796–801. doi: 10.1212/01.wnl.0000049470.00180.07 12629236

[45] Brüggemann N, Mitterer M, Lanthaler AJ, Djarmati A, Hagenah J, Wiegers K, et al. Frequency of heterozygous Parkin mutations in healthy subjects: Need for careful prospective follow-up examination of mutation carriers. Parkinsonism Relat Disord. 2009;15(6):425–9. doi: 10.1016/j.parkreldis.2008.11.014 19162522

