# Supplementary material for: Clinical and genetic characteristics of patients with early-onset or familial parkinsonism: A retrospective single-center cohort
Source: PLoS One. 2026 Oct 27;21(10):e0361198. doi: 10.1371/journal.pone.0361198 (PMC13712704; doi:10.1371/journal.pone.0361198)
Supplement: Supplementary file 1 [file pone.0361198.s001.docx]

**S1 Table. Gene list of the PD-targeted panel**

| Next-generation sequencing (NGS) | | | | | | | |
| --- | --- | --- | --- | --- | --- | --- | --- |
| *ABCB7* | *ADCY5* | *ADH1C* | *AFG3L2* | *ANO10* | *ANO3* | *APTX* | *ATM* |
| *ATN1* | *ATP13A2* | *ATP1A3* | *ATP2B3* | *ATP6AP2* | *ATP7B* | *ATXN1* | *ATXN10* |
| *ATXN2* | *ATXN3* | *ATXN7* | *ATXN8* | *C19orf12* | *C9orf72* | *CACNA1A* | *CCDC88C* |
| *CHCHD2* | *CHMP2B* | *CIZ1* | *COASY* | *COQ2* | *CP* | *CWF19L1* | *DCAF17* |
| *DCTN1* | *DDRGK1* | *DNAJC13* | *DNAJC6* | *DRD2* | *DRD3* | *DRD5* | *ECM1* |
| *EEF2* | *EIF4G1* | *ELOVL4* | *ELOVL5* | *FA2H* | *FBXO7* | *FBXW7* | *FGF14* |
| *FMR1* | *FTL* | *FUS* | *FXN* | *GBA* | *GCDH* | *GCH1* | *GIGYF2* |
| *GJD2* | *GLUD2* | *GNAL* | *GRID2* | *GRM1* | *GRN* | *HAPLN4* | *HMOX1* |
| *HS1BP3* | *HTRA2* | *HTT* | *IFRD1* | *IL1B* | *INPP5F* | *ITPR1* | *KCNC3* |
| *KCND3* | *KCNS2* | *LINGO1* | *LRRK2* | *MAPT* | *MC1R* | *MCCC1* | *NDUFS4* |
| *NOP56* | *NOS3* | *NPC1* | *NR4A2* | *PACRG* | *PANK2* | *PRKN* | *PARK7* |
| *PDYN* | *PINK1* | *PLA2G6* | *PLEKHG4* | *PNKD* | *POLG* | *PPP2R2B* | *PRKCG* |
| *PRKRA* | *PRRT2* | *PSEN1* | *RAB29* | *RAB39B* | *RUBCN* | *SETX* | *SGCE* |
| *SIPA1L2* | *SLC19A3* | *SLC1A2* | *SLC2A1* | *SLC6A3* | *SNCA* | *SNCAIP* | *SNCB* |
| *SORT1* | *SPG11* | *SPR* | *SPTBN2* | *STUB1* | *STX1B* | *SYNE1* | *SYNJ1* |
| *SYT14* | *TAF1* | *TARDBP* | *TBP* | *TDP1* | *TENM4* | *TGM6* | *TH* |
| *THAP1* | *TIMM8A* | *TMEM240* | *TOR1A* | *TOR1B* | *TPP1* | *TRPC3* | *TTBK2* |
| *TUBB4A* | *TWNK* | *UCHL1* | *USP46* | *VPS13A* | *VPS13C* | *VPS35* | *WASF3* |
| *WDR45* | *WWOX* | *ZNF592* |  |  |  |  |  |
| Repeat expansion screening | | | | | | | |
| *NOTCH2* | GGC | | | | | | |
| *NOP56* | GGCCTG/CGCCTG | | | | | | |
| *PHOX2B* | GCN | | | | | | |
| *CACNA1A* | CTG | | | | | | |
| *ATXN2* | GCT | | | | | | |
| *ATXN1* | TGC | | | | | | |
| *TBP* | GCA | | | | | | |
| *ATXN10* | ATTCT | | | | | | |
| *ATXN3* | GCT | | | | | | |
| *AR* | GCA | | | | | | |
| *HTT* | CAG/CCG | | | | | | |
| *FXN* | A/GAA | | | | | | |
| *ATN1* | CAG | | | | | | |
| *ATXN8OS* | CTN | | | | | | |
| *ATXN7* | GCN | | | | | | |
| *DMPK* | CTG | | | | | | |
| *PPP2R2B* | GCT | | | | | | |
| *C9orf72* | GGCCCC | | | | | | |
| *PABPN1* | GCN | | | | | | |
| *FMR1* | CGG | | | | | | |
