# Supplementary material for: Clinical and genetic characteristics of patients with early-onset or familial parkinsonism: A retrospective single-center cohort
Source: PLoS One. 2026 Oct 27;21(10):e0361198. doi: 10.1371/journal.pone.0361198 (PMC13712704; doi:10.1371/journal.pone.0361198)
Supplement: Supplementary file 2 [file pone.0361198.s002.docx]

**S2 Table. Gene list of the Movement disorder (MD) panel**

| Next-generation sequencing (NGS) | | | | | | | |
| --- | --- | --- | --- | --- | --- | --- | --- |
| *AAAS* | *AARS2* | *ABCB7* | *ABCD1* | *ABHD12* | *ACACA* | *ACO2* | *ADAR* |
| *ADCY5* | *ADGRG1* | *ADH1C* | *AFG3L2* | *AHI1* | *AIFM1* | *ALDH18A1* | *ALDH3A2* |
| *ALDH5A1* | *ALG1* | *ALG11* | *ALG12* | *ALG13* | *ALG2* | *ALG3* | *ALG6* |
| *ALG8* | *ALG9* | *ALS2* | *AMACR* | *AMPD2* | *ANO10* | *ANO3* | *AP4B1* |
| *AP4E1* | *AP4M1* | *AP4S1* | *AP5Z1* | *APOB* | *APOE* | *APP* | *APTX* |
| *ARG1* | *ARL13B* | *ARL6IP1* | *ARSA* | *ARSI* | *ARX* | *ASL* | *ASS1* |
| *ATAD3A* | *ATCAY* | *ATG5* | *ATL1* | *ATM* | *ATN1* | *ATP13A2* | *ATP1A2* |
| *ATP1A3* | *ATP2B3* | *ATP2B4* | *ATP6AP2* | *ATP7A* | *ATP7B* | *ATP8A2* | *ATRX* |
| *ATXN1* | *ATXN10* | *ATXN2* | *ATXN3* | *ATXN7* | *AUH* | *B4GALNT1* | *BCKDHA* |
| *BCKDHB* | *BCKDK* | *BCS1L* | *BEAN1* | *BICD2* | *BSCL2* | *BST1* | *BTD* |
| *C12orf65* | *C19orf12* | *C9orf72* | *CA8* | *CACNA1A* | *CACNB4* | *CAMTA1* | *CAPN1* |
| *CASK* | *CAV1* | *CBS* | *CC2D2A* | *CCDC62* | *CCDC88C* | *CCT5* | *CEP290* |
| *CEP41* | *CHCHD10* | *CHCHD2* | *CHMP1A* | *CHMP2B* | *CIZ1* | *CLCN2* | *CLN5* |
| *CLN6* | *CLP1* | *COASY* | *COL18A1* | *COQ2* | *COQ8A* | *COX10* | *COX15* |
| *COX20* | *CP* | *CPLANE1* | *CPS1* | *CPT1C* | *CSF1R* | *CSPP1* | *CSTB* |
| *CTC1* | *CTDP1* | *CTSA* | *CTSF* | *CUL4B* | *CWF19L1* | *CYP27A1* | *CYP2U1* |
| *CYP7B1* | *DAGLA* | *DARS* | *DARS2* | *DBT* | *DCAF17* | *DCTN1* | *DDC* |
| *DDHD1* | *DDHD2* | *DDOST* | *DDRGK1* | *DKC1* | *DLD* | *DNAJC13* | *DNAJC19* |
| *DNAJC6* | *DNM2* | *DNMT1* | *DOLK* | *DPAGT1* | *DPM1* | *DPM2* | *DPM3* |
| *DRD2* | *DRD3* | *DRD5* | *DSTYK* | *DYNC1H1* | *ECM1* | *EEF2* | *EIF2B1* |
| *EIF2B2* | *EIF2B3* | *EIF2B4* | *EIF2B5* | *EIF4G1* | *ELOVL4* | *ELOVL5* | *ELOVL7* |
| *ENTPD1* | *EPM2A* | *ERCC4* | *ERCC6* | *ERCC8* | *ERLIN1* | *ERLIN2* | *ETFDH* |
| *ETHE1* | *EXOSC3* | *EXOSC8* | *FA2H* | *FARS2* | *FAT2* | *FBXO47* | *FBXO7* |
| *FBXW7* | *FGF14* | *FLRT1* | *FLVCR1* | *FMR1* | *FOLR1* | *FOXRED1* | *FTL* |
| *FUS* | *FUZ* | *FXN* | *GAD1* | *GALC* | *GAN* | *GBA* | *GBA2* |
| *GBE1* | *GCDH* | *GCH1* | *GCLC* | *GFAP* | *GIGYF2* | *GJA1* | *GJC2* |
| *GJD2* | *GLB1* | *GLRX5* | *GLUD2* | *GM2A* | *GNAL* | *GOSR2* | *GPNMB* |
| *GPT2* | *GRID2* | *GRM1* | *GRN* | *HAPLN4* | *HEPACAM* | *HEPH* | *HEXA* |
| *HEXB* | *HIBCH* | *HLCS* | *HMOX1* | *HS1BP3* | *HSD17B4* | *HSPD1* | *HTRA1* |
| *HTRA2* | *IBA57* | *IDS* | *IDUA* | *IFIH1* | *IFRD1* | *IL1B* | *INPP5E* |
| *INPP5F* | *ITPR1* | *KCNA1* | *KCNA2* | *KCNC3* | *KCND3* | *KCNJ10* | *KCNS2* |
| *KCTD7* | *KIF1A* | *KIF1C* | *KIF5A* | *KIF7* | *KLC2* | *KLC4* | *L1CAM* |
| *L2HGDH* | *LINGO1* | *LMNB1* | *LRPPRC* | *LRRK2* | *LYST* | *MAG* | *MAN2B1* |
| *MAPT* | *MARS* | *MARS2* | *MC1R* | *MCCC1* | *MECP2* | *MFSD8* | *MIR4697* |
| *MKS1* | *MLC1* | *MMACHC* | *MMADHC* | *MPDU1* | *MPI* | *MPV17* | *MRE11* |
| *MSTO1* | *MTFMT* | *MTHFR* | *MTPAP* | *MTR* | *MTRR* | *MTTP* | *MUT* |
| *MVK* | *NAGS* | *NARS2* | *NDUFA10* | *NDUFAF2* | *NDUFAF6* | *NDUFS3* | *NDUFS4* |
| *NDUFS7* | *NDUFS8* | *NEU1* | *NFU1* | *NGLY1* | *NHLRC1* | *NIPA1* | *NKX2-1* |
| *NOP56* | *NOS3* | *NOTCH3* | *NPC1* | *NPC2* | *NPHP1* | *NR4A2* | *NT5C2* |
| *NUBPL* | *NUCKS1* | *OFD1* | *OPA1* | *OPA3* | *OPHN1* | *OTC* | *PACRG* |
| *PANK2* | *PARK7* | *PAX6* | *PC* | *PCCA* | *PCCB* | *PCLO* | *PDE6D* |
| *PDGFB* | *PDGFRB* | *PDHX* | *PDX1* | *PDYN* | *PEX10* | *PEX16* | *PEX2* |
| *PEX6* | *PEX7* | *PGAP1* | *PGM1* | *PHYH* | *PIK3R5* | *PINK1* | *PLA2G6* |
| *PLD3* | *PLEKHG4* | *PLP1* | *PMM2* | *PMPCA* | *PNP* | *PNPLA6* | *PODXL* |
| *POLG* | *POLR1C* | *POLR3A* | *POLR3B* | *PPP2R2B* | *PRF1* | *PRICKLE1* | *PRKCG* |
| *PRKN* | *PRKRA* | *PRNP* | *PRPS1* | *PRRT2* | *PRX* | *PSAP* | *PSEN1* |
| *PSEN2* | *PTRH2* | *PTRHD1* | *PTS* | *QDPR* | *RAB18* | *RAB29* | *RAB39B* |
| *RAB3GAP1* | *RAB3GAP2* | *RARS2* | *REEP1* | *REEP2* | *RELN* | *RETREG1* | *RFT1* |
| *RIC3* | *RNASEH2B* | *RNF168* | *RNF170* | *RNF216* | *ROGDI* | *RPGRIP1L* | *RRM2B* |
| *RTN2* | *RTN4IP1* | *RUBCN* | *SACS* | *SARS2* | *SCARB2* | *SCN1A* | *SCN8A* |
| *SCTR* | *SDHA* | *SEPSECS* | *SERAC1* | *SETX* | *SGCE* | *SIL1* | *SIPA1L2* |
| *SLC16A2* | *SLC17A5* | *SLC19A3* | *SLC1A2* | *SLC1A3* | *SLC20A2* | *SLC25A1* | *SLC25A13* |
| *SLC25A15* | *SLC25A46* | *SLC2A1* | *SLC30A10* | *SLC33A1* | *SLC52A2* | *SLC6A19* | *SLC6A3* |
| *SLC9A6* | *SNCA* | *SNCAIP* | *SNCB* | *SNORD118* | *SNX14* | *SORT1* | *SPART* |
| *SPAST* | *SPG11* | *SPG21* | *SPG7* | *SPR* | *SPTBN2* | *SQSTM1* | *SRD5A3* |
| *ST8SIA2* | *STK39* | *STT3A* | *STT3B* | *STUB1* | *STX1B* | *SUOX* | *SURF1* |
| *SYNE1* | *SYNJ1* | *SYT14* | *TAF1* | *TARDBP* | *TBC1D20* | *TBP* | *TCTN1* |
| *TCTN2* | *TCTN3* | *TDP1* | *TECPR2* | *TENM4* | *TFG* | *TGM6* | *TH* |
| *THAP1* | *TIMM8A* | *TMEM138* | *TMEM216* | *TMEM230* | *TMEM231* | *TMEM237* | *TMEM240* |
| *TMEM67* | *TOR1A* | *TOR1B* | *TPP1* | *TRAPPC11* | *TRMT5* | *TRNT1* | *TRPC3* |
| *TRPM7* | *TSEN2* | *TSEN34* | *TSEN54* | *TTBK2* | *TTC19* | *TTPA* | *TUBB2A* |
| *TUBB4A* | *TWNK* | *UBE3A* | *UCHL1* | *USP46* | *USP8* | *UVSSA* | *VAMP1* |
| *VARS2* | *VCP* | *VLDLR* | *VPS13A* | *VPS13C* | *VPS35* | *VPS37A* | *VPS53* |
| *VRK1* | *VWA3B* | *WASF3* | *WASHC5* | *WDR45* | *WDR48* | *WDR73* | *WDR81* |
| *WFS1* | *WWOX* | *XK* | *XRCC4* | *ZFR* | *ZFYVE26* | *ZFYVE27* | *ZNF423* |
| *ZNF592* |  |  |  |  |  |  |  |
| Repeat expansion screening | | | | | | | |
| *NOTCH2* | GGC | | | | | | |
| *NOP56* | GGCCTG/CGCCTG | | | | | | |
| *PHOX2B* | GCN | | | | | | |
| *CACNA1A* | CTG | | | | | | |
| *ATXN2* | GCT | | | | | | |
| *ATXN1* | TGC | | | | | | |
| *TBP* | GCA | | | | | | |
| *ATXN10* | ATTCT | | | | | | |
| *ATXN3* | GCT | | | | | | |
| *AR* | GCA | | | | | | |
| *HTT* | CAG/CCG | | | | | | |
| *FXN* | A/GAA | | | | | | |
| *ATN1* | CAG | | | | | | |
| *ATXN8OS* | CTN | | | | | | |
| *ATXN7* | GCN | | | | | | |
| *DMPK* | CTG | | | | | | |
| *PPP2R2B* | GCT | | | | | | |
| *C9orf72* | GGCCCC | | | | | | |
| *PABPN1* | GCN | | | | | | |
| *FMR1* | CGG | | | | | | |
